# London School Children

**Published:** 1906-02-17

**Authors:** 


The Hospital.

A Journal of

The Medical Sciences and Hospital Administration.

Vol. XXXIX.—No. 1,012. SATURDAY, FEBRUARY 17, 1906.

### LONDON SCHOOL CHILDREN.

The valuable report for 1904 of the Medical Officer to the Administrative County of London, to which we have more than once had occasion to refer, perhaps contains nothing more important than the appendix which is devoted to the report of Dr. Kerr, the Medical Officer for the Education Department of the county. This gentleman, who was originally an officer of the School Board, has been retained by the London County Council in its new capacity as the education authority; and he has already been able to set on foot a commencement of the systematic weighing and measuring, coupled with estimation of the capacity for acquiring knowledge, which will have to be conducted for a considerable period as the only trustworthy method of discovering the actual state of the present generation of school children, and of obtaining a standard by which improvement or deterioration may be accurately ascertained. The commencement, so far, is little more than tentative, for the purpose of showing the difficulties to be encountered and the best methods of overcoming them; but Dr. Kerr points out that the children whose measurements will form the units of the inquiry are not interfered with in any way; their self-respect will rather be increased, their sense of belonging to the community heightened by their participating in such measurements, and although the benefit to any individual is scarcely worth considering, yet the results to be hoped for are such that some effort must be made to conduct this inquiry, tedious though it may be to those who do the work.

It seems manifest that, as soon as Dr. Kerr has completely established the best methods of procedure, it will be necessary to relieve him of a task which would seriously interfere with his other duties, and which would be best performed by some organisation brought into existence for the purpose. It is already recorded that Drs. Frances M. D. Berry and C. J. Thomas have examined 1,612 girls and 1,861 boys, and the report gives the results in tabular and diagrammatic form, coupled with classifications based upon apparent poverty, general nutrition, clothing, cleanliness, and intelligence. The diagram shows that the sexes keep very closely together in height and weight up to the thirteenth year, the boys, however, showing some advantage; but after this the natural disparity between them rapidly displays itself. Out of 405 boys in a single school, carefully weighed and measured without boots, the average weight at seven years of age was 19.4 kilogrammes, and the average height 116.5 centimetres; while at 13½ to 14 the average weight was 35.4 kilogrammes, and the average height 141.5 centimetres. These averages were taken from boys, the poverty of whose parents was roughly estimated by the state of the children's clothing; and it was found that 30 boys of the poorest class, possessing each one ragged coat buttoned up, and practically nothing beneath it, and with boots either absent or represented by a mass of rags tied upon the feet, showed considerable failure to reach the averages of height and weight of their age. The average shortage of weight of these boys was as much as 0.7 kilogramme, and the average failure in height a centimetre; 141 boys of a second class, with clothing insufficient to retain animal heat, and needing urgent remedy, and with leaking boots, worked out exactly at the average; while fairly clad boys surpassed it by an average excess of 0.54 kilogramme in weight, and of 1.8 centimetre in height.

There is, therefore, a certain amount of evidence connecting poverty with insufficient development, and so far supporting the belief that it might be of national advantage to adopt some organised method of relieving growing children from its strain. On the evidence at present before him, Dr. Kerr suggests that the excessive shortage of weight in the worst clad children points to the probability that insufficiency of clothing is a definite factor in producing malnutrition, the insufficient food energy being first taxed to keep up the animal heat. Want of cleanliness was found to be almost parallel with want of clothing, both in its presence and in the associated effects; but no correlation could be established between mental capacity and physique. Ninety-three boys below the average in intelligence corresponded with the average age, weight, and height of the school; and the exceptionally brilliant boys (numbering 41) were below the average physique to the extent of 0.25 kilogramme in weight and of 0.4 centimetre in height. It is clear that such investigations, when they have been extended to sufficient numbers and to various localities, will be likely to afford national data of the highest possible value.

